# Identifying opportunities to engage communities with social mobilisation activities to tackle NCDs in El Salvador in the context of the global COVID-19 pandemic

**DOI:** 10.1186/s12939-021-01559-3

**Published:** 2021-10-09

**Authors:** Lizzie Caperon, Stella Arakelyan, Cinzia Innocenti, Alastair Ager

**Affiliations:** 1 Research Unit on Health in Situations of Fragility, Institute for Global Health & Development, Queen Margaret University, Edinburgh, UK; 2 Independent researcher, San Salvador, El Salvador

**Keywords:** Social mobilisation, Non-communicable diseases, Fragile settings, El Salvador

## Abstract

**Background:**

Social mobilisation is potentially a key tool in the prevention of non-communicable diseases (NCDs) in fragile settings. This formative study addressed existing and potential social mobilisation mechanisms seeking behaviour to tackle NCDs in El Salvador, with an emphasis on the implications in the context of the current COVID-19 pandemic.

**Methods:**

We conducted 19 semi-structured interviews with health workers, government officials, NGO leaders, and community members. Interviews addressed mechanisms for social mobilisation which existed prior to COVID-19, the ways in which these mechanisms tackled NCDs, the impact of COVID-19 on social mobilisation activities and new, emerging mechanisms for social mobilisation in the wake of the COVID-19 pandemic.

**Results:**

Findings indicate a growing awareness of NCDs within communities, with social mobilisation activities seen as valuable in tackling NCDs. However, major barriers to NCD prevention and treatment provision remain, with COVID-19 constraining many possible social mobilisation activities, leaving NCD patients with less support. Factors linked with effective social mobilisation of communities for NCD prevention included strong engagement of community health teams within community structures and the delivery of NCD prevention and management messages through community meetings with trusted health professionals or community members. There are gender differences in the experience of NCDs and women were generally more engaged with social mobilisation activities than men. In the context of COVID-19, traditional forms of social mobilisation were challenged, and new, virtual forms emerged. However, these new forms of engagement did not benefit all, especially those in hard-to-reach rural areas. In these contexts, specific traditional forms of mobilisation such as through radio (where possible) and trusted community leaders - became increasingly important.

**Conclusions:**

New mechanisms of fostering social mobilisation include virtual connectors such as mobile phones, which enable mobilisation through platforms such as WhatsApp, Facebook and Twitter. However, traditional forms of social mobilisation hold value for those without access to such technology. Therefore, a combination of new and traditional mechanisms for social mobilisation hold potential for the future development of social mobilisation strategies in El Salvador and, as appropriate, in other fragile health contexts.

## Introduction

Non-communicable diseases (NCDs) are a defining problem of the twenty-first century, hampering nations’ economic growth and sustainable development. Approximately three-quarters (71%) of all-cause global mortality is attributed to NCDs, resulting in 15 million people dying prematurely each year [1]. Cardiovascular diseases, diabetes, cancer and chronic respiratory diseases account for over three-quarters of all NCD associated deaths. They also share key preventable risk factors, namely tobacco use, harmful use of alcohol, unhealthy diets and physical inactivity [1]. The burden of NCDs disproportionately affects low- and middle-income countries (LMICs), where 85% of premature deaths from NCDs occur [1]. The burden of NCDs is further amplified in fragile contexts [2] characterised by exposure to various shocks (e.g., political instability, armed conflict, civil unrest, disasters) and insufficient coping capacity at the state, system or community level to manage and mitigate the effects of these shocks.

El Salvador, a middle-income country in Central America with a population of 6.4 million people [3], exhibits evidence of multiple features distinctive to fragile contexts. The country has a history of brutal violence and armed conflict, and currently has one of the highest homicide rates in the world [4]. The war between local gangs or *maras* is a serious social issue, resulting in extensive population displacements and deaths of thousands each year [4]. El Salvador is being increasingly recognised as the deadliest country in the world outside a war zone with high numbers of violent deaths [5]. NCDs are the main cause of premature mortality among aging Salvadoran population, accounting for 74% of all-caused deaths [1]. Of total deaths registered nationwide, cardiovascular disease, cancer, injuries and diabetes, account for 23, 16, 15 and 5% respectively [1].

Since 2009, the Ministry of Health [MoH] in El Salvador has been implementing a Comprehensive Health System [CHS] reform programme based on Comprehensive Primary Health Care (CPHC) principles: the right to health with equity and solidarity through quality health care provision at the point of delivery [6]. In the 10 years 2009–2018, public health expenditure increased, with two-thirds of public health expenditure (67%) being spent through the public sector and a third through the private sector. First line health coverage was expanded by doubling the number primary care health units to ensure access to primary care in the poorest municipalities. The MoH has embraced and further developed community participation in the preparation, development and management of the CHS reform [7].

Previous studies have explored community and health staff perceptions on NCD management in primary care in El Salvador following the CHS reform [8]. Evidence from these studies suggests that the health system has strengthened its comprehensive approach to NCDs with preventative and community-based strategies. The National Health Forum [NHF], a broad national movement of organised civil society, strongly supported health reform and has encouraged community participation in all the matters affecting their health (Jimenez Carrillo et al., 2020). In 2019, however, a change of government led to uncertainty about the future direction of the health system and how this might impact both NCD services provision and social mobilisation activities led by NHF. These uncertainties have been exacerbated by the COVID-19 pandemic which has strained healthcare systems’ resources and adversely affected clinical decision-making by limiting diagnostic testing for NCDs and physical exams [9].

In March 2020, the El Salvadoran Government imposed restrictions on the use of primary and secondary care services, by cancelling all elective, routine, and non-urgent health care to reallocate resources towards the urgent care of COVID-19 patients [10]. This negatively affected NCD patients’ ability to access care in a timely manner [10]. As the COVID-19 pandemic progresses, there is still considerable uncertainty surrounding the management of individuals affected by NCDs and how existing community-based mechanisms can be used to facilitate social mobilisation activities targeting NCDs [10].

Social mobilisation strategies represent opportunities to communicate, raise awareness and educate at the social level, engage with existing community mechanisms and foster an active partnership between government health agencies and communities [11, 12]. This is particularly important in the context of a global pandemic when fear is heightened and community resources are often a lifeline [13]. Key to these mechanisms in the wake of COVID-19 is a consideration of how communication mechanisms such as virtual platforms can facilitate social mobilisation when face to face social contact is restricted.

We ground this research project in the theoretical understanding of social mobilisation as a tool for the most marginalised and oppressed populations to come together collectively to create change and live healthier lives [14]. Such theoretical approaches emphasise the need for context-specific solutions which challenge ‘one size fits all’ approaches [15]. Such context-specific solutions consider socio-ecological influences such as the socio-cultural environment and political environment on health behaviours [16]. Drawing on previous conceptual frameworks on social mobilisation [17],we assess the components of social mobilisation in the specific context of El Salvador during the COVID-19 pandemic. We undertook this formative research to understand both existing and potential social mobilisation mechanisms for behavioural change to address the growth of NCDs in El Salvador during and following the COVID-19 pandemic.

## Methods

### Data collection and sampling

We conducted 19 semi-structured interviews with health workers, government officials, NGO leaders and community members as shown in Table 1. Purposive, snowball sampling methods were used to recruit participants [18, 19]. We recruited participants who fell within our inclusion criteria for the study and invited them to identify other participants who also fell within these inclusion criteria. Our inclusion criteria were for NGO/Government officials: a) working in an organisation which has involvement in decision-making regarding social mobilisation activities; b) working in an organisation which has been involved in social mobilisation activities before and since the COVID-19 pandemic. Our inclusion criteria for health workers were: a) active in a health setting which has seen social mobilisation activities take place within or connected to it; b) be able to comment on changes in social mobilisation prior to and since the COVID-19 pandemic; and c) involved with treating or supporting individuals with NCDs. Our inclusion criteria for community members were: a) having themselves, or having a close family member with, a diagnosed NCD; and b) living in a community where social mobilisation activities have taken place. Eight health workers were selected from different primary health units serving municipalities with large urban and remote rural populations. Two of the health workers were based in areas with a large indigenous population. We identified officials from key governmental departments and civil society organisations involved in NCD policy implementation and social mobilisation activities. We also interviewed four community members from across the sampled areas.

**Table 1:** Study participants

Gender	Type of Interview	Position
Female	NGO official	NGO Director
Male	NGO official	NGO Director
Female	Government official	Ministry of Health official
Female	Government official	Ministry of Health official
Male	NGO official	NGO Director
Female	NGO official	NGO Director
Female	Government official	Government official
Female	Health worker	Doctor
Female	Health worker	Health promoter
Female	Health worker	Doctor
Male	Health worker	Doctor
Male	Health worker	Doctor
Male	Health worker	Health promoter
Male	Health worker	Medical coordinator
Male	Health worker	Health promoter
Female	Community member	Teacher
Female	Community member	Retired health promoter
Female	Community member	Businesswoman
Female	Community member	Laboratory worker

Interviews were conducted via phone in Spanish by two local researchers experienced in qualitative research. Respondents were contacted at the place of their work or residence and had the project described to them by the researchers. They were then asked if they would like to participate in an interview. If they agreed, a time was organised for this at the participant’s convenience. A participant information sheet and consent form were sent to them via email, and they were asked to complete the form and return it via email to the researcher before the interview. Interviews were structured with a guide consisting of open questions regarding existing social mobilisation activities in communities, potential ways to use social mobilisation in the wake of COVID-19 to support those with NCDs (see Appendix). Interviews with government and NGO representatives additionally addressed capacity within the health system to support people with NCDs through social mobilisation and barriers to providing support to people with NCDs since COVID-19. Interviews lasted for approximately 1 h each.

### Data analysis

Interviews were translated from Spanish into English by the bilingual in-country research team. These were then quality-checked for accuracy by other members of the research team with knowledge of both languages. English summaries of all interviews were collated into an analysis matrix for analysis. After the first three interviews were analysed the research team met to discuss and settle any differences in their analyses. With consistency of the analysis process established, the rest of the data was analysed by the research team with NVivo software using the Framework Approach [20] to allow themes to emerge inductively. The interviews were first coded into the analysis matrix according to the main themes explored in the SSI interview guide. A second round of coding then categorised four major themes which emerged from the analysis. These four themes structure our reporting of results.

### Ethical considerations

The study received ethical clearance from the Queen Margaret University Research Ethics Panel. Officials in the Ministry of Health, El Salvador, granted clearance for the study on the basis of this ethical approval. Prior to each interview, potential participants were telephoned and asked if they would like to take part in the study. If they agreed to take part, an information sheet was provided to each participant to explain the study in detail and all participants were required to sign an informed consent form. Interviews were recorded using a digital voice recorder. Reflective field notes were additionally taken by researchers immediately after completion of the interview. All data were anonymised at the start of data collation. Participation was voluntary and no incentives were given.

## Results

After conducting our analysis using the framework approach, we found our data fit under three distinct themes. These were: (a) experience of NCDs and NCD prevention strategies prior to COVID-19; (b) barriers and facilitators to effective social mobilisation; and (c) impact of COVID-19 on social mobilisation. These three themes are explored in turn.

### Experience of NCDs and NCD prevention strategies prior to COVID-19

There were many existing NCD prevention strategies in the communities of the participants interviewed. In rural communities, Health Committees were considered the main mode for monitoring NCDs. Additionally, community members discussed the ‘Club for the Third Age or Older Adults’, which included people with NCDs such as hypertension and diabetes. In rural areas community meetings were common. Many activities related to health issues were carried out in community gatherings, between relatives and neighbours. Such meetings were important ways of mobilising communities. To be a member of a community committee, such as the health committee, carried with it social status and meant having an important position within the community. Furthermore, in rural areas Family Health Units provided community-based health care with home visits and health promotion activities amongst community members:

*‘ … we as a country have established a model of care based on the family in the community where there are establishments called Family Health Units. These community health teams undertake home visits and foster trust in communities and are aware of how each individual lives within the community. These units know whether individuals comply with medical advice and take medication or not, and how they can guide these patients to improve their awareness of NCDs and prevent them.’ Ministry of Health official*

This policy maker went on to discuss the importance of the family health units in raising awareness of NCDs and the self-care needed to treat them. Political organisation of communities involving various official government structures (e.g., Family health units) and unofficial structures (e.g., civil society organisations) have supported social mobilisation before and since the COVID-19 pandemic.

Our results showed that in the rural population, some level of shame was attached to the diagnosis of NCDs such as diabetes, hypertension among others. Community members stated they did not feel comfortable with the community knowing they were suffering from these diseases:

*‘the term chronic causes stress and tension ... they [patients] know that it is a condition that will be treated for life and they worry about how it will affect their mood and other aspects of their life including relationships with their family and community.’ Ministry of health official*

Recently diagnosed patients stated that they felt shocked to find out that their condition was chronic, and they would have to deal with it for the rest of their lives. Women more frequently engaged with health services than men due to interactions connected to family planning, pregnancy and childcare. Women therefore made up most of the participants on community health committees. Health workers and community members stated that in Salvadorian culture men do not have time to go to health clinics and culturally there was no expectation that men would take care of their health. Health workers reported that sudden deaths from NCD-related complications (e.g., heart attacks or strokes) were far more common in men than women due to untreated/undiagnosed conditions which went undetected due to lower male engagement with health services. One health worker estimated that approximately 70% of males with chronic disease did not attend health consultations to manage their condition.

If there was any male participation in community social mobilisation activities, it was only reported in the ‘clubs for the elderly’, where particular issues related to NCDs were discussed. We found that many NGOs and government institutions focused on empowering women in the communities to help spread health messages:

*‘[ … In our region] the Ministry of Health gives talks on chronic diseases, but we also have help from NGOs that have spent a lot of time in the area (e.g., FUNDESIRA, MEDICOS MUNDI, ADIC, FUSAL, Save the Children). They train community leaders, create Health Committees, provide information about chronic diseases and encourage the spread of health messages by word of mouth. That helped encourage women to become involved in social mobilisation activities led by health units.’ Health worker*

Evidently, the context and experience of NCDs differed between men and women for a range of reasons.

We also found that community members with NCDs discussed challenges around their motivation to improve their dietary behaviour which also impacted on motivation to engage with social mobilisation activities advocating for healthy lifestyle changes. Significant challenges existed relating to accessing healthy food which was often too expensive.

*‘I can give someone a menu [of healthy food] to put it into practice, but the issue is the economic condition, because when it comes to eating apples or pears, how are you going to do so if you don't have money to buy them?’ Health worker*

Health workers stated that in rural areas often those members of the community who produce vegetables, basic grains and fruits, prefer to sell their products instead to leave some for their own consumption (with the exception of corn and beans). This provided a trade-off between nutrition and economic stability in rural communities and often led to greater incidence of NCDs or the worsening of existing conditions. Furthermore, nutrition was a problem in rural areas reliant on coffee plantations where outside of the coffee harvest season people found it difficult to afford more than basic beans and corn for their diet. Health workers reiterated that under such conditions, motivation was hard to generate for NCD prevention through social mobilisation.

### Barriers and facilitators to effective social mobilisation

Several barriers and facilitators were identified which affected engagement in social mobilisation. There was recognition of the need for the government to improve social mobilisation structures in El Salvador and the fact these needs have been heightened by the pandemic:

*‘Since the health reform of 2011 it has been possible to reach places where before there was no access to health ... not that they have been completely successful, but the interest and attention in places where there had previously been none has been vital for some communities. It is important to strengthen the reform and continue to identify new community actors … especially since the pandemic has deepened the precarious conditions that many communities are in.’ NGO Director*

Corroborating this, an MoH coordinator stated that the government works in collaboration with community organisations to tackle NCDs:

*‘Other civil society organizations in the field contribute to the work of MINSAL [Ministry of Health], we have organizations like ASADI (particularly in working with diabetic people) that send health educators to communities to work with NCD patients and promote some self-care projects for them.’ MoH official*

Evidently, some political organisation exists to support communities, though an NGO director stated that social mobilisation activities are not always the priority for the government. He called for the government to work more with key leaders and institutions within communities to bring about change:

*‘There must be an intervention between government institutions and community organisations which recognises the leadership and capacity of people in the community which is essential to achieve a successful mobilisation. You have to work with each community (locality), the churches, the mayors, the governors, the community radios, leaders of the organized civil population.’ Social activist*

This NGO director therefore highlighted the importance of understanding the strong leaders and organisations in communities which can help political efforts and work with the government and political structures to generate mobilisation. There were several political mechanisms which policy makers, health workers and community members discussed as being important including political mechanisms (mayors, governors, civil leaders), religious mechanisms (churches) and others (community radio).

Radio was an important channel of communication for government to tap into to mobilise communities nationwide. Where radio did not exist, health workers and community members stated that communities improvised by using community spaces to transmit health messages to the population. Despite the use of radio, one health worker raised concerns about some rural communities’ access to mobile phone signal and therefore access to information through these methods:

*‘They send [members of the WhatsApp group] messages about the health club, they even send them even a small video chat, but that's here - in the urban area - because here we have mobile signal. But in 80 or 90% of the rural area of ​​the municipality, where the largest number of the population is concentrated, there is no signal … so the online mobilisation strategy does not work there. The health workers who are in the rural area have to visit patients directly [face to face] here it is very difficult to establish other mechanisms than the traditional one or use these technological issues since there is no coverage.’ Health worker*

In rural areas, where greater difficulties were reported in being able to maintain communication via telephone or virtual means, access to resources was limited within families. Health workers reported that it was usually the man who accessed the telephone and controlled its use, and this sometimes represented a barrier to engagement in social mobilisation:

*‘... [communication with individual family members is difficult] what happens is that not all families have a cell phone, and if they have one, the head of the family takes it, does not pass the phone to other members of the family and often won't have it recharged ...’* Health worker

The physical environment had an impact on safety concerns which had knock-on effects on social mobilisation. Concerns were also raised by health workers about physical safety in delivering NCD care due to high crime rates in many areas. Such a context of high crime meant that mobilising communities was dangerous and challenging:

*‘... due to the criminal situation in various communities, the gangs prevented the health promoters from doing awareness-raising and promotion activities and they could not make home visits either ... it is an issue that has already been discussed with the authorities at the level department to find a solution, Health worker*

Social mobilisation activities had clearly been encouraged by NGOs and government institutions, with a focus on women delivering health messages to the community. However, there were important restrictions on women when it came to social mobilisation activities. We found that although women participated in health committees far more than men, and in activities related to health issues in general, they did not have control or management of resources to be able to lead social mobilisation processes. Furthermore, it was stated by health workers that women in indigenous communities often needed to get permission from males in their household to leave their home and attend a health facility, making access to healthcare for any condition, NCDs included, and involvement in any mobilisation activities a challenge for some women.

Facilitators of social mobilisation existed in rural communities led by trusted leaders. Health promotion and health worker training strategies at the primary care level had made it possible to strengthen links between health workers and the communities before COVID-19.

*‘We look for natural leaders and we try to bring them together to form our own Committees that help us … because there are times when we cannot do the work ourselves, and we need the support of the people of the community...’ Health worker*

Community members and health workers both noted that community members had developed trust in informal community structures which included family, peers and neighbours. There was particularly strong trust in the doctors and nurses of the Community Health Teams as well as in peer support networks.

*‘...it is easier for us [community members] to trust those who live in the community … people trust support from their peers. They trust the neighbour who tells their experience with NCDs’ Community member*

Our data suggested that religious leaders represented an important influence in both urban and rural communities and were key in social mobilisation activities; in all territories there were churches and temples, where religious communities meet regularly, which community members and health workers stated had great power and had significant influence on social norms. Community leaders and health workers were also able to transmit messages through churches and community radio stations and often received support from the municipality to do these social mobilisation activities.

Health workers and community members reported that people met in various social spaces which allowed social mobilisation activities to take place. At the community level there were ‘communal houses’, which are spaces used by members of the community that are mostly organised in different types of committees. Other meeting spaces were community leaders’ houses, city halls or schools. These spaces accommodated meetings of smaller groups, when larger groups usually gathered in sports fields and similar large outdoor spaces. Evidently key mobilisers and key mobilisation mechanisms existed before COVID-19 and were able to mobilise communities and support health workers in healthcare delivery.

### Impact of COVID-19 on social mobilisation

Community members reported that the government, and specifically the President, had recently transmitted health messages about COVID-19 to the population through social media channels and wider media (TV, radio). These warned those with NCDs that they were at higher risk of contracting COVID-19. A representative of the MoH stated that such messaging had raised awareness amongst the population about the vulnerability of those with NCDs:

*‘I am convinced that our population is gaining more awareness from this pandemic about the impact of NCDs, because who call the COVID-19 helpline say: ‘I'm hypertensive, I’m worried’ or ‘I'm diabetic’ … there is raised awareness of NCDs as extra risk factors that heighten the risk of complications from COVID-19.’ Policymaker*

This representative stated that many people who call the national helpline about COVID-19 raise concerns about their pre-existing NCDs. Furthermore, it was reported that there was more popular awareness of NCDs and the need to protect vulnerable populations since the COVID-19 outbreak:

*‘People continue to participate [in social mobilisation activities] because they are interested. I think that people's expectations have grown in relation to health issues, there is more vulnerability, people are more fearful and therefore want to be more involved in activities to tackle health issues.’ NGO Director*

During the COVID-19 pandemic the President used national television channels in to communicate his decisions, opinions, policies and health strategies. One MINSAL official told us of alliances that the Ministry had with specific TV channels in the country to deliver health care messages to the general population. Radio was being also actively used to mobilise community responses to the COVID-19 pandemic:

*‘MINSAL [Ministry of Health] is undertaking a national radio campaign in response to COVID-19. Not all communities have access to local radios, they have been instructed to do so through the means they use, for example health personnel are assigned one hour a week in a community space to transmit health messages’ Ministry of Health official*

However, since the COVID-19 outbreak there had been fear around meeting in the physical environment which had impacted on social mobilisation efforts There was recognition that timely detection of NCDs in the population as a whole may reduce as the population is forced to socially distance and refrain from social gatherings:

*‘With all this fear (caused by the pandemic), the confinement could degenerate into Non-Communicable Diseases such as hypertension, cancer … There is a great concern about whether or not individuals belong to a vulnerable population. There is greater need to identify individuals with NCDs or vulnerable to getting them (e.g., those with high blood glucose levels).’ Community organizer*

MoH officials stated that there had been a noticeable reduction in the number of NCD patients accessing health facilities during COVID-19 due to perceived risk of catching COVID-19. This has led to concern about whether NCD patients are receiving the care that they need. Health workers acknowledged that community members took their responsibility of taking care of elderly relatives seriously during COVID-19 and took as many precautions as possible not to bring the disease back to them. This was particularly important in a society where multigenerational households are common and older relatives are socially important in the Salvadoran family structure. Furthermore, as there is no social protection network for the elderly, their families must take care of them.

Since the beginning of the quarantine period due to the COVID-19 pandemic, physical social mobilisation activities were difficult to continue in the country. Community members appeared scared of gathering in large groups and this directly limited any potential social mobilisation activity. Some gatherings had taken place of unions against government actions. Since the outbreak of COVID-19 many of the pre-existing mechanisms to support NCD patients in monthly community-based clubs giving health education had stopped and only some home visits were taking place. This impacted on the amount of support individuals with NCDs had been receiving. One health worker described the potential of reaching these isolated and scared populations through alternative means:

*‘Many of these communities do not have a TV, they do not have a smartphone to read on the Internet, these people receive information best from a radio. However, there are many who do not even radio, word of mouth is an effective way to spread information in these cases. What has to be done is to train community leaders to spread information.’ Health worker*

In communities which do not have access to a TV, or even radio, social mobilisation was primarily by community leaders spreading health messages and mobilising communities to tackle health problems. This channel was prominent following fear associated with the COVID-19 outbreak. Other health workers corroborated the importance of community leaders, particularly those who are part of Health Committees. In the wake of fears about the COVID-19 outbreak it seemed that community members sought information from community leaders they trusted. Prevention measures against COVID-19, such as physical distancing, led to home visits and training of health workers being cancelled.

Our participants stated that social networks had become the main means of social communication for them over the past few years, and this had become intensified since the COVID-19 outbreak. The majority of people reported using WhatsApp and communicating individually or collectively through groups with others on this platform. Twitter was also reportedly popular and the unofficial medium through which the President and the government communicate to the general population. Grassroots health mobilisation groups acknowledged the challenges they now face in having to use more virtual forms of communication to mobilise communities:

*‘Our objectives are the same (as before the pandemic), to improve health in communities. What has changed is the way, the mechanisms to reach people. Because of social distancing, new forms of mobilisation must be used, and everything is generated through communication strategies virtually via social networks. This requires a new way of working that we are not used to.’ NCD Director*

In urban communities in particular, there was more potential for mobilising communities virtually or through social networks such as Facebook, Twitter and WhatsApp and community members in urban areas used these networks regularly to receive health information. However, one MoH official indicated a shift in thinking about the potential support which could be given remotely to NCD patients:

*‘I believe that the pandemic has allowed us to see that we can strengthen ourselves in telemedicine and monitor patients through a telephone call ... or a telecare ... I believe that post-pandemic we must strengthen telemedicine and incorporate it in our policies so that in the future not only NCDs, but also other diseases can be monitored through a call’ MoH Health official*

Additionally, other health workers talked of using social networks more:

*‘So that educational clubs [originally face to face clubs about NCD prevention] keep running, I can send patients messages, I can create groups on social networks or on WhatsApp, where I can send a video in an educational way. We must use other means of education … I can use telephone calls and I can add them to WhatsApp groups to access them. What has to change is the strategy. I must be more proactive now.’ Health worker*

Therefore, in addition to health workers using traditional telephone methods, some had begun to integrate social media and online social networks into their work practice to keep NCD patients engaged with health care. Some community health workers discussed using WhatsApp to forward positive health messages to their patients but cautioned that this information must be checked for reliability and legitimacy, as often community members would believe any WhatsApp messages they received and there was a lot of false information circulating.

In addition to not having mobile phone coverage, some rural areas had been completely cut off during the COVID-19 outbreak:

*‘... the entire rural area in the mountains, is one of the areas with the most difficult access. Access was completely impossible for more than two months; we could not enter and people could not come here...’ Health worker*

In the rural area discussed above by the health worker, there was no phone signal unless you climbed five kilometres up the mountain. Clearly rural populations were facing a myriad of problems accessing health care and using virtual social connectors to mobilise these communities was not always practical.

## Discussion

Our findings indicate that a range of facilitators and barriers exist to engagement in social mobilisation. Such factors are particularly important to consider in fragile health settings like El Salvador where multiple challenges threaten access to health care provision [21]. Examples of facilitators to the engagement in social mobilisation activities we found included the use of virtual social connectors such as WhatsApp. Use of these connectors were socially driven. El Salvador has more mobile phones per person than anywhere in Latin America other than Costa Rica, with penetration rates at 159% [22]. Such mobile phone penetration is changing how people interact and socialise, especially in the wake of the COVID-19 pandemic. Social media has been found to be a powerful tool in mobilising communities [23]. Mobile phone communication networks and platforms represent a clear opportunity to mobilise some communities in El Salvador. However, attention must be paid to developing traditional mobilisation mechanisms (e.g., community-led groups leading NCD prevention activities) in rural communities where a mobile phone signal is often not present. Additionally, those without access to these technologies, such as the elderly and those who cannot afford mobile phones or internet access, must be considered. Social mobilisation activities should be developed to support these groups using existing mechanisms such as radio and TV and community-led groups. Community radio has been found to strengthen social mobilisation in Brazil, Cuba and Haiti [24] as has behaviour change communication [25]; these forms of communication clearly hold the potential to strengthen engagement in social mobilisation in El Salvador.

Those in rural areas were often cut off from others, health care and communication, making mobilisation of the communities difficult to both instigate and coordinate. Those in urban environments had greater access to phone and internet technologies which in turn led to access to virtual social connectors (e.g., WhatsApp). Regardless of location, participants discussed fears connected to high crime rates and risks to their personal safety connected to violence. The remote monitoring and mobilisation of NCD patients discussed by our participants opens a new avenue for potential care and mobilisation, especially during times when patients may be afraid of visiting health facilities such as during times of regional conflict/violence or during health crises such as the COVID-19 pandemic. Social mobilisation represents a strategy for strengthening communities in a context where gang violence is common, as has been found in other countries in the central American region [26].

Our study elicited particularly interesting findings regarding the role of gender in social mobilisation. Participation in social mobilisation was marked by a substantial imbalance between sexes, with gendered norms and structures clearly influential. Our findings indicate that women’s participation is limited by patriarchal structures, with men often having control of resources in the household which enable social mobilisation, such as telecommunications. Women were predominantly involved in health committees and family health is seen as the women’s responsibility due to the importance of maternal and child health. Other research has found that community mobilisation can readdress gender imbalances and limit patriarchal influences such as violence against women [27, 28]. Our findings indicate that such considerations are important to counteract the influence of patriarchal influences in Salvadorian communities and to strengthen female involvement in social mobilisation efforts. However, there also appears to be a need to involve men more in social mobilisation [29]. Social mobilisation can reduce male reluctance to engage in health-related activities and produce more conducive attitudes around involvement in health issues [30].

We found that key community leaders and organisations were important in bringing about social mobilisation in a manner that was trusted by community members, and therefore more likely to be effective. Lessons can be learnt about how to develop such mechanisms to improve social mobilisation. Following the Ebola outbreak in West African in 2016, several studies have investigated the potential of social mobilisation and community engagement to support populations during and after health emergencies in fragile settings [31–33]. These studies appear particularly relevant following the COVID-19 outbreak. Key findings include the need to invest in trusted community members to facilitate community engagement [34]. Considering, adapting to, and drawing on the socio-cultural context appeared to be key from our findings to ensure social mobilisation was effective. Such a consideration of a range of social, political and cultural factors which could influence behaviour, has been found to be key in other fragile health settings [16, 35]. Using key communication networks with wide reach and relevance to the community such as radio and faith-based organisations appeared important, as was the need to invest in strategic partnerships to tap into relevant capacities and resources.

Additionally, our data suggests that COVID-19 prevented the deployment of health teams which had been functioning well in supporting social mobilisation due to trust established with the community. Lack of deployment of health teams had negative impacts on social mobilisation activities. El Salvador could learn from other low-income nations which have successfully deployed health teams in the context of health emergencies to encourage social mobilisation, whether by NGOs or through partnership with international organisations such as the WHO and governments [32]. During the Ebola epidemic in West Africa, the WHO joined with UNICEF to formally embed the communication and social mobilisation component of the Ebola response in Liberia and Sierra Leone [33]. Such strategies could prove to be effective if adapted to the Salvadorian context and endorsed by government and community organisations to tackle some of the inevitable fears associated with COVID-19 and to challenge attitudes of shame associated with NCD diagnoses. Such individual-level factors should therefore be considered in regards to motivation, capability and opportunity to tackle NCDs, with behavioural change techniques holding potential to improve behaviours which lead to NCDs in fragile health contexts [36] in combination with structural interventions.

In the context of El Salvador, the political environment included directives, programmes and initiatives from the government which identified, fuelled and mobilised community leaders and community mobilisation such as the community health committees. Since the 2011 health reform, special emphasis has been placed on primary care, with the strengthening of Community Health Teams communities have been effectively mobilised and Family Health Units further generate awareness of NCDs. These existing political mechanisms were functioning before COVID-19 but needed to be strengthened with further political will and engagement, especially as many of these social methods of mobilisation needed to be reduced or halted completely after the outbreak of COVID-19. Other research has shown an awareness of the potential of social mobilisation to address the increase of NCDs through political structures and there have been recent calls to embed social mobilisation, or civil society mobilisation, into approaches to tackling NCDs [37] with community engagement being advocated as important in addressing NCD prevalence [38]. Such collaboration between government and community organisations can be beneficial. One study explored how social mobilisation could strengthen Brazil’s unified health system [39]. Similar research found the positive impact of social mobilisation on the performance of village health providers in Pakistan [40].

Our study is not without limitations. We were only able to interview a limited number of policymakers, health workers and community members due to budget and time constraints. Our data collection was due to begin in March 2020 before the COVID-19 outbreak; our data collection tools were thus modified to accommodate virtual methods and the number of interviews we were able to conduct were limited by social distancing and government restrictions in El Salvador. Finally, we are aware that we have discussed the political environment impact on social mobilisation mainly from the perspective of policy makers and we did not evaluate all political policies relating to social mobilisation with all types of participants (e.g., community members). We believe, however, that given the constraints of this study, reflections from policy makers and health workers on political issues (e.g., need for more government support to community organisations) were valuable in identifying policies which held the potential to improve social mobilisation.

## Conclusion

Our findings indicate a growing awareness of NCDs within communities, with social mobilisation activities helping to tackle NCDs. Factors such as gender played a key role in social mobilisation activities, with societal norms influencing involvement based on traditional gender roles. However, major barriers to NCD prevention and treatment provision remain, with COVID-19 further constraining activities. New, virtual forms of social mobilisation are rapidly emerging, but need to be complemented by traditional forms of mobilisation - such as radio and through trusted community leaders - given limited accessibility to technology within poorer households and in hard-to-reach rural areas.

## Data Availability

Not Applicable.

## Appendix

### Interview guides [translated from Spanish]

The following questions were asked to the policymakers:

1. Do you deliver any health interventions on national level? Local level?
2. How have these interventions changed since COVID-19?
3. Which existing structures (e.g. communication networks, radio channels etc.) are in place to facilitate interventions that involve engagement between government/public authorities and communities?
4. Have these structures been used during COVID-19? How?
5. Does intersectional working (across government sectors/departments) take place to engage communities? if so, how?
6. Which social mobilisation activities have occurred BEFORE COVID-19? What have these been used to tackle? Have any of these activities addressed NCDs?
7. Has COVID-19 lead to changes in social mobilisation activities? If yes, how?
8. What are the implications of social distancing and quarantine measures on social communication and social mobilisation?
9. What is needed to make social mobilisation interventions successful?
10. Who is involved in successful social mobilisation initiatives?
11. Who do community members trust? How does this effect their engagement with health services?
12. How are topics are chosen for existing interventions? (are needs assessments conducted? To what extent does funding [where from] influence interventions?)
13. Too what extent do you think community needs for health care are being met?
14. What have been the barriers and facilitators for intervention success?
15. How do you think existing structures could be used more to inform potentially effective, sustainable and scalable approaches to delivering NCD interventions?
16. What support have you provided for people with co-morbidities/NCDs during the COVID-19 pandemic?
17. How could these vulnerable people be better supported?
18. Has there been any change in awareness about the problem of NCDs since the COVID-19 pandemic? If yes, which changes have been seen?
19. How has the COVID-19 pandemic changed people’s perception of health?
20. Does evaluation take place of existing interventions? If so, how? If evaluation does not take place, how could evaluation mechanisms be developed?

The following questions were asked to the community leaders/health workers:

1. Which social mobilisation activities have occurred BEFORE COVID-19? What have these been used to tackle? Have any of these activities addressed NCDs?
2. Has COVID-19 lead to changes in social mobilisation activities? If yes, how?
3. What are the implications of social distancing and quarantine measures on social communication and social mobilisation?
4. How are you involved in social mobilisation activities?
5. How has your involvement changed since COVID-19?
6. What is needed to make social mobilisation interventions successful?
7. Who is involved in successful social mobilisation initiatives?
8. Who do community members trust? How does this effect their engagement with health services?
9. How are topics are chosen for existing interventions? (are needs assessments conducted? To what extent does funding [where from] influence interventions?)
10. Too what extent do you think community needs for health care are being met?
11. What have been the barriers and facilitators for intervention success?
12. What support have you provided for people with co-morbidities/NCDs during the COVID-19 pandemic?
13. How could these vulnerable people be better supported?
14. Has there been any change in awareness about the problem of NCDs since the COVID-19 pandemic? If yes, which changes have been seen?
15. How has the COVID-19 pandemic changed people’s perception of health?
16. Does evaluation take place of existing interventions? If so, how? If evaluation does not take place, how could evaluation mechanisms be developed?

The following questions were asked to the community members:

1. Were you involved in any social mobilisation activities BEFORE COVID-19? What were these activities? Did any of these activities addressed NCDs?
2. Has COVID-19 lead to changes in social mobilisation activities? If yes, how?
3. What are the implications of social distancing and quarantine measures on social communication and social mobilisation?
4. What is needed to make social mobilisation interventions successful?
5. Who is involved in successful social mobilisation initiatives?
6. Who do you trust and how does who you trust effect your involvement in social mobilisation activities?
7. Too what extent do you think community needs for health care are being met?
8. What have been the barriers and facilitators for intervention success?
9. Has there been any support for people with co-morbidities/NCDs during the COVID-19 pandemic?
10. How could these vulnerable people be better supported?
11. Has there been any change in awareness about the problem of NCDs since the COVID-19 pandemic? If yes, which changes have been seen?
12. How has the COVID-19 pandemic changed you perception of health?

## References

[1] WHO. Noncommunicable diseases. 2021 [cited 2021 May 6]. Available from: https://www.who.int/news-room/fact-sheets/detail/noncommunicable-diseases

[2] Atun RA, Bennett S, Duran A (2008). When do vertical (stand-alone) programmes have a place in health systems? [internet].

[3] World Bank. Renovating the Public Health Care System in El Salvador. 2019 [cited 2021 May 6]. Available from: https://www.worldbank.org/en/results/2019/04/25/renovating-the-public-health-care-system-in-el-salvador

[4] World Bank. Intentional homicides (per 100,000 people) - El Salvador [Internet]. 2018 [cited 2021 May 6]. Available from: https://data.worldbank.org/indicator/VC.IHR.PSRC.P5?locations=SV

[5] Lakhani N. Violent deaths in El Salvador spiked 70% in 2015, figures reveal. The Guardian [Internet]. 2016 [cited 2021 May 6]; Available from: https://www.theguardian.com/world/2016/jan/04/el-salvador-violence-deaths-murder-2015

[6] Ministry of Health El Salvador. Technical guidelines for family ECOs and specialized ECOs; 2017.

[7] Barten F, Rovere M, Espinoza E (2010). Reforma de salud: un contexto adverso para el desarrollo de la atención primaria de salud integral en El Salvador.

[8] Jimenez Carrillo M, León Garciá M, Vidal N, Bermúdez K, De Vos P (2020). Comprehensive primary health care and non-communicable diseases management: a case study of El Salvador. Int J Equity Health.

[9] Lagarde M, Masferrer MS, Herl CR. El Salvador’s COVID-19 response is storing up health and economic problems for the worse-off [Internet]. LSE Blog. 2020 [cited 2021 May 6]. Available from: https://blogs.lse.ac.uk/latamcaribbean/2020/10/16/el-salvadors-covid-19-response-is-storing-up-health-and-economic-problems-for-the-worse-off/

[10] World Bank. El Salvador aborda la principal causa de muerte prematura entre su población adulta [Internet]. 2021 [cited 2021 May 6]. Available from: https://www.worldbank.org/en/news/feature/2021/03/22/el-salvador-aborda-causa-de-muerte-prematura-poblacion-adulta

[11] Public Health England. The community response to coronavirus (COVID-19) [Internet]. Public Health Matters Blog. 2020 [cited 2020 Oct 8]. Available from: https://publichealthmatters.blog.gov.uk/2020/06/01/the-community-response-to-coronavirus-covid-19/

[12] Tony Blair Institute for Global Change. COVID-19 Social Mobilisation in Disease Outbreaks: Principles, lessons and examples from the Ebola response in Sierra Leone. London; 2020.

[13] Marston C, Renedo A, Miles S (2020). Community participation is crucial in a pandemic. Lancet..

[14] Freire P (1970). Pedagogy of the oppressed.

[15] Campbell C. Community mobilisation in the 21st century: Updating our theory of social change?: http://dx.doi.org/101177/1359105313500262. 2013 Sep 2 [cited 2021 Aug 13];19(1):46–59. Available from: https://journals.sagepub.com/doi/10.1177/1359105313500262 10.1177/1359105313500262 24000384

[16] Caperon L, Arjyal A, Puja KC, Kuikel J, Newell J, Peters R (2019). Developing a socio-ecological model of dietary behaviour for people living with diabetes or high blood glucose levels in urban Nepal: A qualitative investigation. PLoS One.

[17] Pettifor A, Lippman SA, Gottert A, Suchindran CM, Selin A, Peacock D, et al. Community mobilization to modify harmful gender norms and reduce HIV risk: results from a community cluster randomized trial in South Africa. J Int AIDS Soc. 2018;21(7). 10.1002/jia2.25134 PMC6058206 29972287

[18] Palinkas LA, Horwitz SM, Green CA, Wisdom JP, Duan N, Hoagwood K (2015). Purposeful sampling for qualitative data collection and analysis in mixed method implementation research. Adm Policy Ment Health.

[19] Green AE, Aarons GA (2011). A comparison of policy and direct practice stakeholder perceptions of factors affecting evidence-based practice implementation using concept mapping. Implement Sci.

[20] Gale NK, Heath G, Cameron E, Rashid S, Redwood S (2013). Using the framework method for the analysis of qualitative data in multi-disciplinary health research. BMC Med Res Methodol.

[21] Diaconu K, Falconer J, Vidal N, O’May F, Azasi E, Elimian K, et al. Understanding fragility: Implications for global health research and practice. Health Policy and Planning. 2020;35:235–43 Oxford University Press. Available from: pmc/articles/PMC7050687/. Accessed 14 Aug 2021. [cited 2021 May 6] 10.1093/heapol/czz142 PMC7050687 31821487

[22] Molina K. El Salvador es el segundo en Latinoamérica con más líneas móviles que personas. Noticias de El Salvador [Internet]. 2019 [cited 2020 Oct 8]; Available from: https://www.elsalvador.com/noticias/negocios/el-salvador-es-el-segundo-en-latinoamerica-con-mas-lineas-moviles-que-personas/568522/2019/

[23] Wang J, Madnick S, Li X, Alstott J, Velu C. Effect of Media Usage Selection on Social Mobilization Speed: Facebook vs E-Mail. PLoS One [Internet]. 2015;10(9):e0134811. Available from: http://ovidsp.ovid.com/ovidweb.cgi?T=JS&PAGE=reference&D=med11&NEWS=N&AN=26422171 10.1371/journal.pone.0134811 PMC4589319 26422171

[24] RM dos S G, V de C O (2015). International cooperation Brazil-Cuba-Haiti: the role of community radios in strengthening social mobilization in the public health context in Haiti. Cien Saude Colet.

[25] Wasan PG. POLIO eradication: overview of social mobilization through behavior change communication mix and interpersonal communication in India. J Serv Res. 2015;15(1) unpaginated. Available from: http://jsr.vedatya.ac.in/adsmin.asp?Details=284.

[26] Middeldorp N, Morales C, van der Haar G (2016). Social mobilisation and violence at the mining frontier: the case of Honduras. Extr Ind Soc.

[27] Treves-Kagan S, Maman S, Khoza N, MacPhail C, Peacock D, Twine R, et al. Fostering gender equality and alternatives to violence: perspectives on a gender-transformative community mobilisation programme in rural South Africa. https://doi.org/101080/1369105820191650397. 2019 Apr 20 [cited 2021 Aug 13];22(sup1):127–44. Available from: https://www.tandfonline.com/doi/abs/10.1080/13691058.2019.1650397 10.1080/13691058.2019.1650397 PMC7905832 31429663

[28] Minckas N, Shannon G, Mannell J. The role of participation and community mobilisation in preventing violence against women and girls: a programme review and critique. https://doi.org/101080/1654971620201775061. 2020 Dec 31 [cited 2021 Aug 13];13(1). Available from: https://www.tandfonline.com/doi/abs/10.1080/16549716.2020.1775061 10.1080/16549716.2020.1775061 PMC7480621 32588783

[29] Rebombo D. One man can-as a community mobilisation strategy; 2016. https://www.svri.org/sites/default/files/attachments/2016-07-05/Community%20Mobilisation%20as%20a%20VAW%20Prevention%20strategy.pdf. Accessed 14 Aug 2021.

[30] Pettifor A, Lippman SA, Gottert A, Suchindran CM, Selin A, Peacock D, et al. Community mobilization to modify harmful gender norms and reduce HIV risk: results from a community cluster randomized trial in South Africa. J Int AIDS Soc. 2018;21(7) Jul 1 [cited 2020 Oct 8];Available from: /pmc/articles/PMC6058206/?report=abstract. 10.1002/jia2.25134 PMC6058206 29972287

[31] Pedi D, Gillespie A, Bedson J, Jalloh MF, Jalloh MB, Kamara A (2017). The development of standard operating procedures for social mobilization and community engagement in Sierra Leone during the West Africa Ebola outbreak of 2014–2015. Spec Issue Commun community Engagem response to Ebola, 2014–2015. Evid lessons Futur Glob Heal Cris.

[32] Gillespie AM, Obregon R, El Asawi R, Richey C, Manoncourt E, Joshi K (2016). Social Mobilization and Community Engagement Central to the Ebola Response in West Africa: Lessons for Future Public Health Emergencies. Glob Heal Sci Pract.

[33] Laverack G, Manoncourt E (2016). Key experiences of community engagement and social mobilization in the Ebola response. Glob Health Promot.

[34] Arakelyan S, Jailobaeva K, Dakessian A, Diaconu K, Caperon L, Strang A, et al. The role of trust in health-seeking for non-communicable disease services in fragile contexts: A cross-country comparative study. Soc Sci Med. 2021; Under rev. 10.1016/j.socscimed.2021.114473 PMC8689406 34662762

[35] Caperon L, Brand-Correa L (2020). Sustainable electricity for sustainable health? A case study in North-Western Zambia. J Energy South Africa.

[36] Caperon L, Sykes-Muskett B, Clancy F, Newell J, King R, Prestwich A (2018). How effective are interventions in improving dietary behaviour in low- and middle-income countries? a systematic review and meta-analysis. Health Psychol Rev.

[37] Heller O, Somerville C, Suggs LS, Lachat S, Piper J, Aya Pastrana N, et al. The process of prioritization of non-communicable diseases in the global health policy arena. Health Policy Plan. 2019; Available from. 10.1093/heapol/czz043. 10.1093/heapol/czz043 PMC6736081 31199439

[38] Amminadab J (2019). First-ever WHO global report on epilepsy highlights care gap in poorer countries | UN News. UN News.

[39] Fernandes VR, ZP da L, AC de A, Sergio JV, JPV da S, MC e CM M (2017). The “locus” of health oversight in Brazil’s Unified Health System - a place between the knowledge and the practices of social mobilization. Spec Issue Surveill Heal Soc right to Heal Promot Prot.

[40] Gine X, Khalid S, Mansuri G. The impact of social mobilization on health service delivery and health outcomes: evidence from rural Pakistan. Policy Res Work Pap - World Bank. 2018;(8313):23 Available from: https://openknowledge.worldbank.org/bitstream/handle/10986/29223/WPS8313.pdf.

